# Can Priming Legal Consequences and the Concept of Honesty Decrease Cheating During Examinations?

**DOI:** 10.3389/fpsyg.2019.02887

**Published:** 2020-01-21

**Authors:** Yu-Wei Wu, Lu-Lu Zhong, Qian-Nan Ruan, Jing Liang, Wen-Jing Yan

**Affiliations:** ^1^Student Affairs Division, Wenzhou Business College, Wenzhou, China; ^2^Institute of Psychology and Behavior Sciences, Wenzhou University, Wenzhou, China; ^3^School of Educational Sciences, Ludong University, Yantai, China

**Keywords:** examination cheating, priming paradigm, honesty conception, legal consequences, field study

## Abstract

Cheating on exams is a very common phenomenon that causes great harm. Various measures, such as chastisement and direct punishment, have been employed to reduce cheating. Previous studies have found that increasing punishment and activating “self-concept maintenance” can reduce this behavior. This study employed a priming paradigm to investigate whether priming legal consequences and the concept of honesty would reduce cheating in examination situations. In experiment 1, a total of 402 freshmen from 17 classes were included in this study. The 185 students in experimental condition were primed for legal consequences. The cheating behaviors and employed analysts were defined to count the number of cheaters. The results show that the number of students cheating in the primed group did not decrease compared to those in the controlled condition. In experiment 2, a total of 386 freshmen from 16 classes participated in this experiment. The 171 students in experimental condition were primed for the concept of honesty. The results also show that the number of students cheating in the primed group did not decrease. This study shows that priming legal consequence and the concept of honesty were not significant in certain situations, such as during examinations. It is suggested that some psychological manipulations in decreasing dishonesty behaviors should be further tested in ecological situations.

## Introduction

The history of cheating on exams is as long as the process of examination itself. Some scholars have described cheating in universities as something that is as natural as breathing, an academic skill as important for college students as reading, writing, and mathematics (Moffatt, 1990). It has been said that the belief that “everyone’s doing it” is not far from the truth (Cizek, 1999; Jensen et al., 2002). The proportion of students who cheat on examinations is high; 59% of high school students admitted to cheating on a test in the past year [Josephson Institute (2010) ethics report card 34% indicated that they had cheated more than once]. Nearly half of the students queried admitted to cheating at least occasionally on tests (Galloway, 2012).

The harm caused by cheating on exams is obvious. It has been argued that the experience of successfully cheating might reinforce cheating behaviors (Mccabe and Trevino, 1993; Cizek, 1999; Landry, 2003), making students more likely to cheat in the future. Cheating also puts honest students at a disadvantage. This is why schools have taken various measures to prevent cheating on exams. Yet, cheating continues to be an issue. Before we try to develop more appropriate measures to reduce cheating, we must screen the mechanisms by which students cheat.

### Theories on Cheating

Based on rational assumptions in rational economics, it has been argued that rational beings will make decisions that maximize profit, based on a rational analysis of the particular situation (Louden, 2000). Some researchers have combined benefits and risks in rational economics to study cheating behaviors (Smolensky et al., 1968; Sandmo, 2005). In fact, to bring in a steady stream of income, individuals tend to pursue long-term positions of maximum benefit. For example, an individual might believe that he is lucky enough to avoid being caught by the police for deliberately illegally parking. Based on the actions of rational beings, researchers have proposed that dishonest behaviors could be reduced by increasing the risk of being caught, increasing the associated penalty, and reducing the level of reward. Students may be tempted to cheat if the punishment from being caught is not as severe as the potential benefit of passing the examination and receiving a high score (Jawahar et al., 2007; Ma et al., 2013). For example, a poor student might cheat on an examination if the consequences of failing are more severe than the risk of cheating. After all, a simple glance at someone else’s answers might help them pass the exam. However, it is human nature to be sensitive to risks, which is called risk aversion according to prospective theory (Kahneman and Tversky, 1979). With potential risks of being caught, students should be serious to make a decision of cheating. Therefore, it is reasonable that reminding the risks of being caught right before the exams should restrict their cheating behaviors.

In addition, being social animals, humans must regulate their behavior and maintain their self-image. They have principles they follow when pursing their own interests, except for when pursuing the maximization of profits. For some, even if the benefits are attractive and abundant, they will not abandon their principles. In recent years, researchers such as Ariely and Gino have shown that our deception decisions do not always follow completely rational assumptions; individuals often pay more attention to the effects of a self-concept than profit and risk, at least with regards to cheating behaviors (Gino et al., 2011; Gino and Ariely, 2012). These researchers proposed the “self-concept maintenance theory,” arguing that our deceptive behaviors could be the result of weighing the possible profits against maintaining a particular self-concept (de Quervain et al., 2004). Self-concept maintenance theory holds that people may cheat to maximize self-profit, but only to the extent that they can do so while maintaining a positive self-concept. An individual profiting from cheating may affect their ability to retain a positive self-concept and thus influence the deception (Liang et al., 2016). We are told to be honest and want to be considered honest, which means that the wise engage in moral caution (i.e., what cannot be done, what can be done, and what qualities we should have) when we are tempted to cheat.

### Priming Restrictions or Positive Virtues as a Means of Reducing Cheating

Studies have found that priming people’s restrictions based on what should not be done (i.e., religious tenets) and encouraging positive self-concepts such as the acceptance of oneself as a person with positive virtues might reduce dishonest behaviors. Ariely explored the impact of religious priming on cheating, based on the knowledge of common ethical guidelines (such as the Ten Commandments). The research split 450 participants into two groups and asked half to try to recall the Ten Commandments. They were then tempted to cheat on a matrix task. The other half were asked to try to recall 10 books they had read in high school, before being given their matrices and the opportunity to cheat. The results showed that, in the second group, there typically was widespread but moderate cheating. Conversely, there was no cheating in the group in which participants were asked to recall the Ten Commandments, despite no one in the group being able to recall all 10 (Ariely, 2012).

Randolphseng and Nielsen (2007) also examined the impact of religious priming on dishonesty. Before the experiment, participants were provided with chaotic sentences. These sentences contained references to religion, sport, and a number of neutral words. Individuals were then asked to arrange the words into grammatically correct sentences. By arranging the words related to religion, the subject’s religious tenets were primed. In the experiment, respondents completed a circle task, closing their eyes to write numbers in a small circle. The researchers set unreachable goals and high rewards, tempting them to lie. The reward was determined by the performance of the circle task. It was found that participants in the religious priming group were far less likely to cheat than those in the other two groups. The results showed that when individuals’ religious tenets were primed, they consciously abided by those tenets and the frequency of dishonest behavior was reduced (Randolphseng and Nielsen, 2007). The above research suggests that it is the effort to maintain a self-concept that reduces cheating, via adherence to various regulations. Some researchers have also studied other aspects of self-concept maintenance theory as a motivation to maintain an individual’s positive moral image (i.e., exhibit honesty) and thus reduce cheating.

Research has indicated that people generally value honesty and a strong sense of morality and want to maintain a positive self-concept (Greenwald, 1980; Sanitioso et al., 1990; Griffin and Ross, 1991; Smyth and Davis, 2004). Mazar et al. (2008) found that when students were required to sign an honor code, they were less likely to act in an untrustworthy manner; this is in line with individuals’ preference for honesty and self-morality and also can be adjusted to accommodate subsequent dishonest behavior (Wheeler and Berger, 2007; Sela and Shiv, 2009). Studies have also found that signing at the top of such a code can serve as an ethical reminder and thus be more effective than signing at the bottom. The reason is that signing at the top may constrain behavior after it is tested, while signing at the bottom has no effect on previous behavior (Shu et al., 2012).

Based on previous research, it seems that cheating could be reduced by (1) increasing the associated penalty based on rational assumptions and (2) inspiring a positive self-image based on self-concept maintenance theory. Moreover, restrictions or positive virtues could be primed by simple manipulations. For the former, legal consequences are a restriction (i.e., an exogenous factor) that may limit people’s behaviors. According to the hypothesis of rational beings, being aware of the potential risks should reduce bad behavior. Specifically, in our experiment, an actual examination situation, we used legal consequences as the restriction because, in some countries (such as China), individuals do not have strong religious tenets. For the latter, priming honest is a direct and proved to be effective method to reduce the cheating behaviors, and we could ask them to sign their names below a statement of honesty.

Two experiments in an actual examination context were carried out. Fortunately, there are students with the same major in a university who were traditionally with extraordinarily proportion of cheating in examinations. It facilitates our research in an ecological situation. Experiment 1 evaluated whether priming the legal consequences of cheating before an exam would reduce cheating. Experiment 2 considered whether priming the concept of honesty through signing a promise to adhere to an honor code would reduce examination dishonesty. It was hypothesized that there would be fewer students cheating after they were primed with legal consequences and the concept of honesty than in the controlled condition. The psychological mechanisms of cheating were analyzed, and empirical research on how to reduce students’ cheating behaviors in daily life was provided.

## Experiment 1

### Method

Before beginning, we informed the Student Affairs Office of the purpose of our research and obtained permission to conduct the experiment and collect video recordings. The participants were recorded with their full knowledge. Each examination room was set up with a camera capable of covering the entire room to prevent cheating. Before the examination week, students were instructed that they were under inspection by the camera and warned of the risk of getting caught. In addition, each inspector was told the day before for the examination to request their assistance. The classroom cameras were confirmed to be installed and operational.

### Participants

This study was conducted at a regular university in China. A total of 402 freshmen from 17 classes were studied in experiment 1, while 217 students from 9 classes were randomly set in the controlled condition. Another 185 students from eight classes were set randomly in the experiment condition (i.e., primed for legal consequences). These participants were from the same major, and the students of that major historically did not follow the examination rules well. None of the students in this experiment were aware of its purpose. The examination process is under monitoring by cameras according to school rules. The experiment was approved by the Institutional Review Board.

### Materials

#### Materials Provided to Students

The experiment classes were provided with handouts listing the legal consequence of cheating on examinations, as outlined in “Criminal Law” and “Teachers’ Law” (see Supplementary Appendix I). The participants were, to a large extent, becoming teachers. These articles were chosen because the students were relatively familiar with them, and most could relate to them. I appreciate the reviewer’s suggestion that the content might be slightly complicated. If it is difficult for students to read through the handout, they might simply skim it. Then, inspectors asked the students to read and sign the handouts because we thought this would make them take the process seriously and carefully read the content. The controlled classes were provided with blank forms; all students in each classroom were asked to sign their names to indicate their attendance. The presentation of these specific legal consequences primed their risk awareness.

#### Materials Provided to Inspectors

In the controlled condition (see Supplementary Appendix II), the inspectors conducted the procedures required by the university. For the legal priming condition, the inspectors distributed handouts, listing the legal consequences of cheating to each student and asked that each student sign their name. The inspectors were provided with step-by-step instructions and made sure that each followed the same process (see Supplementary Appendix III for details).

### Procedure

**FIGURE 1:**
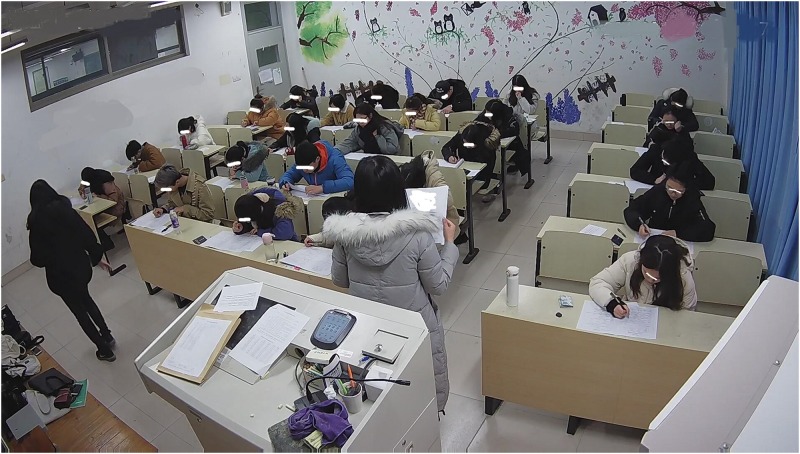
Students inside an exam room.

All researchers gathered in an office half an hour before starting the experiment and obtained the experiment materials for the class for which they were responsible. They then waited for the exam inspectors outside the respective classrooms. When each inspector arrived, the associated researcher provided them with the experiment materials. All researchers waited outside their assigned classrooms during the experiment, in case there were any problems. All examination rooms were equipped with video cameras that clearly captured the examination process. Figure 1 illustrates the exam environment.

### Data Collection and Analysis

(1) Seventeen videos from the monitoring control office were collected.
(2) Manual coding was used for the collected videos. Thirty-four students majoring in psychology (and thus were from a different department) were recruited to analyze the videos, with each person analyzing two videos. Thus, each video had two different coders so that reliability could be accurately calculated. We needed 34 coders because of the substantial burden of coding each recording.
(3) The computer room were arranged, and the video players were installed beforehand. All coders were gathered together in the computer room. The coders were prohibited from taking photos or engaging in any other acts that would violate students’ privacy.
(4) Since individuals have different levels of sensitivity to cheating behaviors, we asked an experienced teacher to provide suggestions for common cheating techniques and defined some types of cheating (see Supplementary Appendix IV). The researchers distributed the coding sheets (see Supplementary Appendix V) and gave instructions on how to properly complete them.
(5) These 34 (17 × 2) videos were distributed to the 17 analysts. Each individual was required to analyze two different videos; thus, there were two coding sheets for each video. At the end of the analysis, there were 34 coding sheets finished. Two or three coders would have achieved better reliability, but the workload would have been too heavy, there were numerous videos of the examination rooms that needed to be coded. For better reliability, the coders were trained beforehand and established similar sets of criteria. Such manipulations are a tradeoff between reliability and efficiency.
(6) The coders watched their videos and recorded any cheating students (according to seat number), as well as the time and method of the cheating (see Supplementary Appendix V for details). To motivate colleagues to carefully search for cheaters, coders who found fewer cheaters than their partners were asked to review the recording again. In the review session, they verified their partners’ coding and discussed any disagreements. This allowed us to identify if there was any disagreement between coders and facilitate subsequent arbitration. Finally, the coders reported the total number of people who cheated in each video. The intercoder correlation is 0.41. There will be a discussion for the low reliability in section “Discussion.”
(7) To motivate the colleagues to carefully search for the cheaters, the coders who found fewer cheaters than their partner were asked to review the recording again. In cases of non-conformity (usually resulting from missed targets), these coders could identify the position and time of the cheat. Through this process, each video eventually produced the necessary data, and all coding for all videos was completed.

### Results

Based on the arbitrated coding data, the number of cheating and non-cheating students in the controlled and legal consequences conditions are tabulated and arranged in Table 1.

**TABLE 1:** Total cheating and non-cheating students in different conditions.

	Cheating	Non-cheating
Controlled condition	106	111
Legal consequences condition	95	90

The information collected for this study was in the form of enumeration data; therefore, an independent test was employed in the non-parametric test.

A chi-square comparing the number of cheaters in the controlled condition to that of the legal consequences condition was not significant, χ^2^(1, *N* = 402) = 0.25, α = 0.05, *p* = 0.617, effect size = 0.05, *1* − *β* = 0.079. A sensitivity analysis were conducted using G^∗^Power (Faul et al., 2007) and found that, given α = 0.05, a power of *1* − *β* = 0.95 and *N* = 402, we could detect differences between the group with the reminders and the control group as small as *w* = 0.18. This effect size is between small (*w* = 0.1) and medium (0.3) effects in terms of the effect size conventions introduced by Cohen (2013). The results showed that there was no significant difference in the tendency toward truthfulness between the controlled (51.2%) and legal consequences (48.6%) conditions. The number of cheating students in the legal consequences condition was less than in the controlled condition. This implies that priming legal consequences had no effect on reducing students’ cheating.

In experiment 1, priming legal consequences (exogenous restrictions) did not reduce cheating. It may be due to the little effect of priming an exogenous restriction in such a situation, or it may be because of not well-manipulated experiment such as the two complicated description of legal consequences in the handout for priming. It is difficult for the students to read through the handout but might just have a browse.

It is possible, on the other hand, that inspiring the concept of honesty (inner positive virtue) may help to reduce the cheating behaviors.

## Experiment 2

### Method

The preparations before the experiment were the same as those of experiment 1.

### Participants

A total of 339 freshmen from 16 classes participated in this experiment; all were in the same final exams. There were two conditions in experiment 2. In the first, 168 students from seven classes were distributed randomly in the controlled condition; in the second, 171 students from seven classes were distributed randomly in the experiment condition (i.e., priming the concept of honesty). None of the students were aware of the experiment’s purpose or the monitoring cameras. The experiment was approved by the Institutional Review Board. We hypothesized that there would be less cheating in the experiment condition priming honesty than in the controlled condition.

### Materials

#### To Students

Controlled classes were provided with attendance forms; experiment classes were provided with a handout specifying the concept of honesty (see Supplementary Appendix VI).

#### To Inspectors

For the controlled condition, see Supplementary Appendix II; for the experiment classes, see Supplementary Appendix VII.

### Procedure

The procedure was the same as that which was employed in experiment 1.

### Data Collection and Analysis

The process of data analysis in experiment 2 was the same as that which was employed in experiment 1. Fourteen videos were collected after the experiment, and 16 people were recruited to analyze them; each person analyzed two videos, meaning that each video had two different coders. The processes for training and coding were the same as those employed in experiment 1. At the end of the data analysis, we received 28 data sheets. The intercoder correlation is 0.40. Next, persons finding fewer cheaters than their colleagues for the same video were required to analyze the video again to verify the total number of cheaters. Through this process, 14 sets of data were obtained.

### Results

The experimenters reviewed the numbers of cheating and non-cheating students and summarized the numbers of each for the controlled and honesty conditions. The final data are shown in Table 2.

**TABLE 2:** Total cheating and non-cheating students in different conditions.

	Cheating	Non-cheating
Controlled condition	86	82
Honesty condition	86	85

The information collected for this study was in the form of enumeration data; therefore, an independent test in the non-parametric test were employed.

A chi-square comparing the number of cheaters in the controlled condition to that of the honesty condition was not significant, χ^2^(1, *N* = 339) = 0.028, *p* = 0.866, with effect size 0.009, α = 0.05, *1* − *β* = 0.054. A sensitivity analysis were conducted using G^∗^Power (Faul et al., 2007) and found that, given α = 0.05, a power of *1* − *β* = 0.95 and *N* = 339, we could detect differences between the group with the reminders and the control group as small as *w* = 0.20. This effect size is between small (*w* = 0.1) and medium (0.3) effects in terms of the effect size conventions introduced by Cohen (2013). The results showed that there was no significant difference in the tendency toward truthfulness between the controlled (51.2%) and honesty (50.2%) conditions. This implies that priming the concept of honesty in internal psychology had no obvious effect on reducing students’ cheating.

## Discussion

The results of these experiments indicate that there is no significant difference between either the honesty or legal consequences conditions and the controlled condition. In other words, priming legal consequences, and honesty both failed to reduce cheating in college students in the examination situation. These results seemed to reject the hypotheses that priming restrictions and referencing virtue will reduce dishonesty, as has been argued in previous research (Mazar et al., 2008). Possible reasons for this difference are as follows.

First, participants in many studies receive only small rewards for (or simply enjoy) taking part in psychological experiments (Mazar et al., 2008; Gino and Mogilner, 2014), and any rewards received are not essential. In our study, the students were strongly motivated to pass the exam because failing could lead to their retaking the course, the loss of a scholarship, criticism from their parents, etc. When the possibility of profit is strong enough, the priming effects of legal consequences and the concept of honesty are not significant. As for risk, students seemed to believe that even if they cheated during a test, as long as the infraction was not too serious, the college would generally not punish them too severely. It was also difficult for supervisors to provide evidence of someone cheating; as a result, many turned a blind eye to the behavior. Therefore, even though students’ sense of honesty and recognition of legal consequences were stimulated, they still cheated because the benefits far outweighed the risks. Moreover, whether cheating behavior is shown is controlled by the severity of the negative consequences multiplied by the probability of being caught. Given this, if the subjective probability of being caught is considered closed to zero, then even severe consequences will not have an effect. As previous research found, juveniles’ decisions tend to be motivated more by rewards rather than risks (Steinberg and Scott, 2003). Moreover, it is true that historically, very few people had been caught and severely punished, or the punishment was much less severe than what was being claimed.

Second, participants in the work of Mazar et al. had no opportunity to prepare for the tasks following the reference to their sense of morality. Allowing participants time to recognize that they might profit from their actions could serve to exaggerate the numbers. In our experiments, the students planning to cheat on their exams had time to fully prepare (such as by producing notes in advance), so priming before the examination was unlikely to change their minds.

The third reason could be a tendency to imitate cheating peers. In previous studies of this type of behavior (Mazar et al., 2008), the participants were strangers to one another, and there was no direct competition among them. When the task was over, the researcher’s feedback did not create additional pressure on them. In the current study, the students were all from the same class; final grades would determine their rankings. Even if students read the letter of commitment and signed their name or recognized the negative outcomes of cheating, they may have felt that it would offer their classmates an unfair advantage if they refrained from cheating while others continued to do so. Thus, pressure from their peers’ cheating could have motivated them to behave similarly (Treviño et al., 1998; Schubert et al., 2010).

The forth reason could be that the tutors educated them to be honest and warn them of the severe consequences of being caught before the examination week. In other words, the students clearly understood the legal consequences and social expectations. However, it is true that, to some degree, the examination situation already primed these legal consequences and the concept of honesty, although this was several days before the behavior.

The above-mentioned reasons may explain why there were the significant results of the manipulation to reduce the cheating behaviors. Although the insignificance is due to various reasons including not well-manipulated experimental design, it also raised the caution to apply the priming techniques, no matter with exogenous factor (legal restrictions) or endogenous factors (self-image of being honest). Our study echoed another Registered Replication Report describing the aggregate result of 25 direct replications (total *n* = 5,786), all of which followed the same preregistered protocol. In the primary meta-analysis (19 replications, for a total of *n* = 4,674), participants who were given the opportunity to cheat reported solving 0.11 more matrices if they were given a moral reminder than if they were only given a neutral reminder (95% CI: −0.09, 0.31) (Verschuere et al., 2018). This minor effect was numerically in the opposite direction to that of the original study (Cohen’s *d* = −0.04); thus, priming had no effect, a result consistent with our study.

At last, there are some words on the reliability—the interrater reliability is 0.41 for experiment 1 and 0.40 for experiment 2. The low reliability is partly due to the large number (more than 20) of students in the examination room, which makes the situation very complicated. There may have been inconsistencies in labeling time and location. Therefore, it was very difficult to reach high agreement on both time and location. However, cheaters were seldom treated unjustly. Mostly, the inconsistency was due to neglect. To remedy this, one of the coders were asked to check the missing values and any arbitrary disagreements.

## Conclusion

This study attempted to test whether priming legal consequences and honesty would reduce students’ cheating behaviors. However, consistent with some research in this area, such priming was found to have no effect. Therefore, the priming of legal consequences and honesty will not always reduce cheating, at least in situations such as examinations. It is suggested that some psychological manipulations in decreasing dishonesty behaviors should be further tested in ecological situations.

## Data Availability Statement

The raw data supporting the conclusions of this article will be made available by the authors, without undue reservation, to any qualified researcher.

## Ethics Statement

The studies involving human participants were reviewed and approved by the Institutional Review Board in Institute of Psychology and Behavior Sciences in Wenzhou University. The patients/participants provided their written informed consent to participate in this study.

## Author Contributions

W-JY and Y-WW conceived and designed the experiments. Y-WW, L-LZ, and Q-NR performed the experiments. L-LZ, JL, Q-NR, and W-JY analyzed the data. W-JY, Y-WW, and L-LZ wrote the manuscript. Y-WW and JL revised the manuscript.

## Conflict of Interest

The authors declare that the research was conducted in the absence of any commercial or financial relationships that could be construed as a potential conflict of interest.
