# Supplementary material for: Can Priming Legal Consequences and the Concept of Honesty Decrease Cheating During Examinations?
Source: Front Psychol. 2020 Jan 21;10:2887. doi: 10.3389/fpsyg.2019.02887 (PMC6985441; doi:10.3389/fpsyg.2019.02887)
Supplement: Supplementary file 1 [file Data_Sheet_1.docx]

Appendix Ⅰ

According to “**The Criminal Law of the Peoples Republic of China**,” Article 284, “**Whoever unlawfully uses any special equipment or devices for eavesdropping or secret photographing**, if the consequences are serious, shall be sentenced to fixed-term imprisonment of not more than two years, criminal detention, or public surveillance.”

Article 284a reads: “**Whoever engages in cheating in any national examination** … shall be sentenced to fixed-term imprisonment of not more than three years or criminal detention and concurrently or separately sentenced to a fine if the circumstances are serious; the person shall be sentenced to fixed-term imprisonment of not less than three years but not more than seven years and concurrently sentenced to a fine.

**Whoever provides devices for cheating or other forms of assistance allowing others** to commit the crime prescribed in the preceding paragraph shall be punished in accordance with the provisions of the preceding paragraph.

**Whoever illegally sells or provides to others the examination questions or answers** for examinations prescribed in Paragraph 1 in order for others to cheat on an examination shall be punished in accordance with the provisions of Paragraph 1.

**Whoever takes another’s name or has another person take his name in an examination** prescribed in Paragraph 1 shall be sentenced to criminal detention or public surveillance and concurrently or separately sentenced to a fine.”

**According to “Teachers’ Law of the People's Republic of China**,**”** Article 14, “Those who have been deprived of political rights or subjected to fixed-term imprisonment or even more severe punishment for intentional crimes **shall not be allowed to obtain qualification as a teacher; those who have already obtained the qualification of teacher shall forfeit such qualification.**”

Name:

Student ID:

Date:

Appendix Ⅱ

**The procedure for the baseline condition:**

1. The investigator will check the classroom to determine whether students have made cheat sheets or left cheat materials in desks or other places prior to the examination.

2. Please follow the seating chart on the blackboard and do not independently change seats.

3. Provide a signal table list for students to fill in and then distribute the test papers.

4. Please carefully check students’ numbers, seat numbers, and other personal information. During the examination period, if you find that students have cheated on the exam, please confront them in private.

5. At the end of the test, researchers will uniformly recover all experiment materials.

Appendix Ⅲ

**Legal consequences condition:**

1. The investigator will check the classroom to determine whether students have made cheat sheets or left cheat materials in desks or other places prior to the examination.

2. Please follow the seating chart on the blackboard and do not independently change seats.

3. Provide the guarantee and ask students to sign their name. Then read the following words:

Hello everyone! Recently, a new requirement was added in which students must read a guarantee and sign their name. After signing yours, please put it on the corner of the desk. Thank you for your cooperation.

4. Distribute the test papers.

5. Please carefully check students’ numbers, seat numbers, and other personal information. During the examination period, if you find that students have cheated on the exam, please confront them in private.

6. At the end of the test, researchers will uniformly recover all experiment materials.

Appendix IV

The experienced inspectors provided us some suggestions of the cues of cheating:

1. Sitting in a strange posture (backward, recline);
2. Looking at the inspectors frequently;
3. Stiff left arm;
4. Holding the fist;
5. Paying attention to the bottom of the pencil case and water glass;
6. Stopped writing after choice question session;
7. Those who began to look around often just after several minutes and usually do this until the end of the exam;
8. Those in the front row with his back to the back table and trying to whisper;
9. Trying to look at other people's papers;
10. To sum up, those who didn’t focus on their own papers during the exam are suspicious;

The following were the typical cheating behaviors:

1. Looking at something (usually a small piece of paper) in their palms;
2. Looking at something (usually a small piece of paper) in their sleeves;
3. The front and back students whispering;
4. Looking at others’ papers for some time (and afterwards continued writing).
5. Other behaviors that are obviously against the examination rules.

Appendix V

Analyzer:（ ） Classroom：（ ）

Notes:

1. Coordinates represent students’ test seats.

(X, Y): X represents the column of the student’s seat, which counts from the left of the classroom next to the door, and Y represents the row of the student’s seat, with the first row next to the classroom’s blackboard.

1. If you have trouble deciding whether or not someone is cheating, please add "?" so that we can recheck the video later.
2. When you are recording the time of the cheating, please refer to the time in the upper right-hand corner of the video.

For example:

| Serial number | Time | Event (please specify the coordinates of the cheater and indicate his cheating behavior) |
| --- | --- | --- |
| 1 | 00:30:00 | Peeked at others’ answer |
| 2 | 00:35:12 | Peeked at cheat sheet |
|  |  |  |
|  |  |  |
|  |  |  |
|  |  |  |
|  |  |  |

Total number of cheaters ( )

Appendix VI

Student ID:

Honesty is a traditional virtue of the Chinese. (Name) promises to be an honest person, and will not cheat in the following examination.

Date:

Appendix VII

1. The investigator will check the classroom to determine whether students have made cheat sheets or left cheat materials in desks or other places prior to the examination.

2. Please follow the seating chart on the blackboard and do not independently change seats.

3. Provide the guarantee and ask students to sign their name. Then read the following words:

Hello everyone! Recently, a new requirement was added in which students must read a guarantee and sign their name. After signing yours, please put it on the corner of the desk. Thank you for your cooperation.

4. Distribute the test papers.

5. Please carefully check students’ numbers, seat numbers, and other personal information. During the examination period, if you find that students have cheated on the exam, please confront them in private.

6. At the end of the test, researchers will uniformly recover all experiment materials.
